# Intraoperative flexible nephroscopy during percutaneous nephrolithotomy: An 8 years’ experience

**DOI:** 10.12669/pjms.37.3.3565

**Published:** 2021

**Authors:** Yasir Masood, Nadeem Iqbal, Raja Mohsin Farooq, Sajid Iqbal, Faheemullah Khan

**Affiliations:** 1 Yasir Masood Resident Urology, Shifa International Hospital, Islamabad, Pakistan; 2 Nadeem Iqbal Urology, Shifa International Hospital, Islamabad, Pakistan; 3 Raja Mohsin Farooq Rehbar Medical and Dental College, Lahore, Pakistan; 4 Sajid Iqbal Department of Rehabilitation, PNS Hospital, Karachi, Pakistan; 5 Faheemullah Khan Resident Diagnostic Radiology, Aga Khan University Hospital, Karachi, Pakistan

**Keywords:** PCNL, Stones, Flexible Nephroscopy, Urolithiasis, Staghorn

## Abstract

**Objectives:**

To see the effect of intra operative antegrade flexible nephroscopy during Percutaneous nephrolithotomy on stone free rate.

**Methods:**

We retrospectively reviewed electronic medical records of patients who underwent percutaneous nephrolithotomy from 2010 to 2017 for renal stones >2cm. Patients found eligible were divided in, Group-I who did not have intraoperative Flexible nephroscopy and Group-II who had flexible nephroscopy during percutaneous nephrolithotomy. All procedures were done by senior consultants. Variables like Mean age, side, stone size, skin to stone distance and Hounsfield unit were compared. Outcomes like Stone free rate, hospital stay and operative time were compared between the groups.

**Results:**

The study included 248 patients, consisting 85 (34.3%) females and 163 (65.7%) males. Mean age ± SD was 45.8±13.8 years. Both group were similar in characteristics like mean age, stone size, skin to stone distance and Hounsfield units. The overall stone free rate was 71%. It was not significantly different between the groups, 76% in Group-II vs. 67% in Group-I. However stone free rate markedly improved with flexible nephroscopy in patients with staghorn calculi. Mean operative time and hospital stay were similar between the groups.

**Conclusions:**

Intraoperative flexible nephroscopy during percutaneous nephrolithotomy significantly increases stone free rate in patients with staghorn stones.

## INTRODUCTION

Renal stones are a common pathology with annual prevalence of 2-3% worldwide.^1^ Pakistan is located in the middle of the Afro-Asian stone belt, with stone prevalence of 12–15%. Stone disease comprise of 50% of urological workload in adults, while 60% in children.^2^ Percutaneous nephrolithotomy (PCNL) represents the standard of treatment for renal stones >2cm. It is a minimally invasive procedure providing high success rates with an excellent safety profile.^2^-^6^

Intraoperative detection of residual fragments during PCNL may be challenging. Presence of residual fragments may need for auxiliary procedures like ESWL, flexible URS or redo PCNL.^5^-^9^ Flexible nephroscopy is another modality being used in different centers to improve the outcomes of PCNL. However, there is a wide variability in literature regarding the efficacy of intraoperative flexible nephroscopy in PCNL.

In this study we wanted to evaluate the role of intraoperative flexible nephroscopy during PCNL on stone free rate and other outcomes like operative time and hospital stay.

## METHODS

We retrospectively reviewed the electronic medical record of patients undergoing PCNL, for renal stones >2cm, from 2010 to 2017, after the approval of institutional review board and ethical committee (IRB#1126-402-2018). Total 248 patients above age 12 were found eligible. Patients without pre-op non contrast CT scan, no post op imaging and requiring nephrostomy tube were excluded from this study. All patients had pre-operative non contrast CT scan and underwent PCNL under general anesthesia in prone position. All procedure were done by two senior consultants (ten years of experience).

Initially 6Fr ureteric catheter was placed into the renal pelvis in supine position through 22Fr rigid cystoscope to delineate pelvicalyceal system. Then patient was placed in prone position with all pressure points adequately covered. Subcostal puncture was done into appropriate calyx with 18Fr Chiba needle under fluoroscopy guidance. 0.035inch glide wire inserted into the upper tract via Chiba needle sheath. Tract was dilated with metallic coaxial dilators up to 24 or 27Fr followed by placement of 26 or 30Fr amplatz sheath. Rigid nephroscope 24Fr (Richard Wolf GmbH, Knittlingen) was used in all cases. Stones were broken with pneumatic lithoclast and removed with 3-prong grasper. Stone clearance was achieved using fluoroscopy with rigid nephroscope or flexible nephroscope 16.5Fr (karl-storz). All patients had 6Fr JJ stent (Boston Scientific, US). Patients were given antibiotic coverage during hospital stay. All were discharged on 1^st^ or 2^nd^ post-operative day. Stone free status was determined on follow-up with x-ray or ultrasound KUB within 02 weeks of procedure. Residual stones <4mm were considered stone free. JJ stents were removed after achieving stone free status.

Patients were divided in two groups. Group-I had 116 patients without flexible nephroscopy and Group-II had 132 patients with flexible nephroscopy. The decision whether to perform flexible nephroscopy was based on surgeons preference, dictated by fluoroscopic imaging showing any opacities in renal area or stone configuration on imaging, where residual fragments are likely to be present. Both group were reviewed for gender, side, stone size, skin to stone distance, Hounsfield units, operative time, hospital stay, and stone free status. Stone size was calculated in largest diameter in mm^2^ by multiplying maximum length and max width. Point starting from cystoscopy for insertion of ureteric catheter to the point of skin suturing of the PCNL tract was taken as operative time. Skin to stone distance was calculated via pre op CT scan in lateral view by measuring distance between skin to the stone in the kidney. Mean Hounsfield units were calculated by selecting a 1cm^2^ area in the center of the stone. All data were collected on a specified Performa.

IBM SPSS statistics version 25 was used for data analysis. Mean ± standard deviation was calculated for quantitative variables like age, stone size, skin to stone distance, Hounsfield units, operative time and hospital stay. Chi Square test was used to compare stone free rates and their significance. P value < 0.05 was considered significant.

## RESULTS

This study included 248 patients, consisting 85 (34.3%) females and 163 (65.7%) male with female to male ration of 0.52. Mean age ± SD was 45.8±13.8 years (range 15-79). Left to right side ratio was 1.4:1. Group-I, included 77 male (66%), with mean age of 46.1+13.9 years vs. 45.5+13.8 years in Group-II, which included 86 males (65%). There was no significant differences among these groups regarding the age and gender ratio (Table-I). Both group were similar in characteristics like mean age, mean stone size, mean skin to stone distance and mean Hounsfield units (Table-I). All procedures were done via single tract approach. Surgeons preference and stone location were the determining factors as to the pole of entry (lower pole, upper pole or mid pole).

**Table-I:** Demographic characteristics

Characteristics	Group-I	Group-II	p-value
Number of patients (n)	116	132	
Male/females (n)	77/39	86/46	
Mean age ± SD (years)	46.1±13.9	45.5±13.8	0.727
Mean tract length (mm)	88.9	88.4	0.717
Mean stone size (mm^2^)	764	781	0.805
Mean Hounsfield units	1190	1074	0.078
No. of access	1	1	

Overall stone free rate was 71%. Patients in Group-II had better stone free rate 76% vs. 67% in patients Group-I, however this was not statistically significant (p=0.137). Mean operative time in Group-I was slightly less, 118 minutes vs. 124 minutes in Group-II (p=0.345). Even though, operative time was longer in Group-II but it did not result in prolongation of hospital stay or the complications. Mean hospital stay in both groups were insignificantly different. (Table-II).

**Table-II:** Outcomes of PCNL.

Outcomes	Group-I	Group-II	p-value
Stone free (percentage)	78 (67%)	100 (76%)	0.137
Operating time (min)	118	124	0.345
Hospital stay (mean)	3.09	3.07	0.849
Staghorn calculi (n)	31	29	
Stone free (percentage)	11(35%)	22(76%)	0.002
Operative time (min)	126.9	156.7	<0.01
Hospital stay (mean)	3.39	3.17	0.259

Patients among both groups were further stratified according to presence of staghorn calculi. Total 60 patients had staghorn calculi. Among these patients who underwent flexible nephroscopy had significantly better stone free rate 76% vs. 35% (p= 0.002) as compared to patient who didn’t have flexible nephroscopy. There was insignificant difference in mean hospital stay in both groups (p=0.259). Mean operative time in Group-II was significantly longer 156.7 vs. 126.9 minutes (p<0.01).

## DISCUSSION

Ever since PCNL was first introduced in 1980s, many advancement in technique and instrumentation have occurred further improving the efficacy and reducing morbidity.^10^ PCNL has been the standard of care for renal stone >2cm giving high success rates.^11^ Flexible nephroscopy is utilized by number of centers to enhance the outcomes of PCNL however there has been no consensus on the usage of flexible nephroscopy in stone clearance after PCNL. American urological association also recommends to perform flexible nephroscopy routinely after PCNL.^12^

We did not find any significant difference in stone free rates in our patients who had standard PCNL vs. PCNL with flexible nephroscopy. Goktug et al, also found no significant effect of flexible nephroscopy on overall stone free rate in PCNL in his retrospective review of 1250 renal stone patients including 166 staghorn calculi patients. Stone free rate was 87% with flexible nephroscopy vs. 81% without it.^13^ In a similar study by Desai et al, a retrospective analysis of 684 patients who underwent PCNL with multiple tracts or PCNL with flexible nephroscopy through single tract. They found no difference in stone free rate among both groups.^14^ On the other hand Gucuk et al, in their randomized controlled trial of 80 patients, found better stone free rate with intraoperative flexible nephroscopy 92.5% as compared to without flexible nephroscopy 70% (p=0.02).^15^

In patient with staghorn stones, our study found an excellent outcome with the use of flexible nephroscopy in stone free rate. Goktug et al, in their study found to have better stone free outcome in staghorn patients with the usage of flexible nephroscopy with PCNL via single tract approach. They had mean operating time of 95 and 113 minutes for standard PCNL and PCNL with flexible nephroscopy respectively.^13^

Marguet et al, advocated the use of flexible instrument in their study by combining PCNL with flexible ureteroscopy in decreasing the need of multiple tract and increasing stone free rate in patients with complex renal calculi,^16^ however this requires simultaneous access via retrograde route and others have tried approach of utilization of expensive flexible ureteroscopes. Akman et al, retrospectively reviewed 413 patient with staghorn calculi underwent PCNL with flexible nephroscopy via single or multiple tract approach, reported stone free rate of 70.1% for PCNL with flexible nephroscopy via single tract.^17^ Recently Sfoungaristos et al, published a study in which they retrospectively reviewed 103 patients with staghorn stones treated by PCNL with flexible nephroscopy via single tract approach over the period of 10 years, reported a stone free rate of 65%,^18^ much similar to our results.

Second look nephroscopy has also been advocated in literature to achieve better stone clearance. Wong and Leveillee had their study of 49 patients managed using second look flexible nephroscopy after PCNL under general anesthesia or sedation, after acquiring post op CT scan, for renal stones >5cm.^19^ this resulted in better stone free outcome but on expense of a second general anesthesia. El-Nahas et al published a study on staghorn stones with PCNL and reported stone free rate of 56% overall and 65% (p=0.01) in 146 patients who underwent PCNL with flexible nephroscopy via single tract approach.^20^

Instead Knudsen advocated the use of aggressive flexible nephroscopy at the time of initial PCNL for reducing the need for second-look procedures, which needed nephrostomy tube followed by post op flexible nephroscopy in outpatient clinic or in operating room under general anesthesia/sedation.^21^ Roth et al, shared his experience of PCNL in 24 children who were managed by second look nephroscopy 48 to 72 hours postoperatively to achieve complete stone clearance.^22^

Our study did not find significant difference on the overall stone free rate, mean operative time and hospital stay with the usage of flexible nephroscopy. However, it significantly improved stone free rate in patients with staghorn calculi on the expense of little increase in operative time. Hence we would strongly recommend flexible nephroscopy especially in patients with staghorn calculi.

### Limitations of the study:

It is a retrospective in nature, no randomization, lack of clear criteria as to which patients were chosen for flexible nephroscopy and short follow up. However, it had its strength as it was a first study of its kind in Pakistan.

## CONCLUSION

The use of intraoperative antegrade flexible nephroscopy improves stone free rate after PCNL, significantly for staghorn calculi. We would recommend its routine use in such cases to minimize the need of ancillary procedures.

### Authors’ Contribution:

**YM, NI, SI** conceived, designed and did statistical analysis & editing of manuscript.

**YM, SI, FK, RMF,** did data collection and manuscript writing.

**YM, NI** takes the responsibility and is accountable for all aspects of the work in ensuring that questions related to the accuracy or integrity of any part of the work are appropriately investigated and resolved.

## References

[1] Shohab D, Iqbal N, Alam MU, Butt A, Jamil MI, Hussain I (2016). Comparison of Outcome of Percutaneous Nephrolithotomy in Adult Versus Paediatric Patients. J Coll Physicians Surg Pak.

[2] Rizvi SA, Hussain M, Askari SH, Hashmi A, Lal M, Zafar MN (2017). Surgical outcomes of percutaneous nephrolithotomy in 3402 patients and results of stone analysis in 1559 patients. BJU Int.

[3] Iqbal N, Hasan A, Siddiqui FS, Iftikhar F, Siddiqui FS, Ilyas SM (2019). Outcome of Percutaneous Nephrolithotomy in Preschool and School-Age Children-Single Center Experience. J Ayub Med Coll Abbottabad.

[4] Yang W, Cui Z, Ma T, Zhao C, Zhou H, Guo J (2018). Effects of visual standard channel combined with visual superfine precision puncture channel or super-mini channel percutaneous nephrolithotomy on multiple renal calculi. Pak J Med Sci.

[5] Aquil S, Rana M, Zaidi Z (2006). Laparoscopic assisted percutaneous nephrolithotomy (PCNL) in ectopic pelvic kidney. J Pak Med Assoc.

[6] Yang L, Lu S, Han X, Wei P, Yang J, Hao T (2016). Clinical comparison of the efficiency and security of balloon dilators versus fascial dilators in percutaneous nephrolithotripsy (PCNL). Pak J Med Sci.

[7] Malik HA, Tipu SA, Mohayuddin N, Sultan G, Hussain M, Hashmi A, Naqvi SA, Rizvi SA (2007). Comparison of holmium:Yag laser and pneumatic lithoclast in percutaneous nephrolithotomy. J Pak Med Assoc.

[8] Samad L, Zaidi Z (2012). Tubed vs tubeless PCNL in children. J Pak Med Assoc.

[9] Iqbal N, Hasan A, Malik HA, Khan R, Nazar A, Khawaja MA (2020). A Comparison of Complications and Success Rates after PCNL in Younger and Elderly Patients. J Coll Physicians Surg Pak.

[10] Kim BS (2015). Recent advancement or less invasive treatment of percutaneous nephrolithotomy. Korean J Urol.

[11] Lim SH, Jeong BC, Seo SI, Jeon SS, Han DH (2010). Treatment outcomes of retrograde intrarenal surgery for renal stones and predictive factors of stone-free. Korean J Urol.

[12] Assimos D, Krambeck A, Miller NL, Monga M, Murad MH, Nelson CP (2016). Surgical Management of Stones:American Urological Association/Endourological Society Guideline. J Urol.

[13] Goktug G, Karakoyunlu N, Sener NC, Zengin K, Nalbant I, Karabacak O (2015). Standard percutaneous nephrolithotomy alone versus in combination with intraoperative anterograde flexible nephroscopy for staghorn stones:A retrospective study. Kaohsiung J Med Sci.

[14] Desai M, Ganpule A, Manohar T (2008). “Multiperc”for complete staghorn calculus. J Endourol.

[15] Gucuk A, Kemahli E, Uyeturk U, Tuygun C, Yildiz M, Metin A (2013). Routine flexible nephroscopy for percutaneous nephrolithotomy for renal stones with low density:A prospective, randomized study. J Urol.

[16] Marguet CG, Springhart WP, Tan YH, Patel A, Undre S, Albala DM (2005). Simultaneous combined use of flexible ureteroscopy and percutaneous nephrolithotomy to reduce the number of access tracts in the management of complex renal calculi. BJU Int.

[17] Akman T, Sari E, Binbay M, Yuruk E, Tepeler A, Kaba M (2010). Comparison of outcomes after percutaneous nephrolithotomy of staghorn calculi in those with single and multiple accesses. J Endourol.

[18] Sfoungaristos S, Mykoniatis I, Katafigiotis I, Isid A, Gofrit ON, Constantinides CA (2018). Single lower calyceal percutaneous tract combined with flexible nephroscopy:A valuable treatment paradigm for staghorn stones. Canadian Urolog Assoc J.

[19] Wong C, Leveillee RJ (2002). Single upper-pole percutaneous access for treatment of >5-cm complex branched staghorn calculi:is shockwave lithotripsy necessary?. J Endourol.

[20] El-Nahas AR, Eraky I, Shokeir AA, Shoma AM, El-Assmy AM, El-Tabey NA (2012). Factors affecting stone-free rate and complications of percutaneous nephrolithotomy for treatment of staghorn stone. Urology.

[21] Knudsen BE (2009). Second-look nephroscopy after percutaneous nephrolithotomy. Ther Adv Urol.

[22] Roth CC, Donovan BO, Adams JM, Kibar Y, Frimberger D, Kropp BP (2009). Use of second look nephroscopy in children undergoing percutaneous nephrolithotomy. J Urol.

